# Major fires in Indonesian Borneo are possible under all ENSO phases

**DOI:** 10.1038/s44304-026-00209-4

**Published:** 2026-04-23

**Authors:** Timothy Lam, Jennifer L. Catto, Gillian Kay, Nick Dunstone, Rosa Barciela, Anna B. Harper

**Affiliations:** 1 Centre for Doctoral Training in Environmental Intelligence, University of Exeter, Exeter, UK; 2 Faculty of Environment, Science, and the Economy, University of Exeter, Exeter, UK; 3 Met Office, Exeter, UK; 4 Department of Geography, University of Georgia, Athens, GA USA; 5 Present Address: WCRP Regional Information for Society (RIfS), Montreal, QC Canada

**Keywords:** Climate sciences, Natural hazards

## Abstract

Major fires in Indonesian Borneo over the past few decades caused devastating impacts on human health, livelihoods, economy, the natural habitat and the carbon sink. Despite the proven link between the positive phase of the El Niño Southern Oscillation (ENSO) and rainfall deficit, a primary driver of fires, the rare occurrence of historical major fires makes it difficult to robustly assess their climatic cause using purely the observational record. We analysed a large initialised ensemble of global climate simulations to demonstrate that although the risk of severe fires that level or surpass those observed in 1997 and 2015 is strongly boosted by an El Niño by a factor of 2.7, such risk exists regardless of the ENSO phase. Other factors modulating the risk include the Indian Ocean Dipole (IOD) and a Rossby wave train over the southern extratropics which acts to decouple the rainfall response from the ENSO state. Our study highlights that even in places characterised by high seasonal predictability, there is still a possibility of high impact weather events to occur against the expected response to a given teleconnection mode, which implies the necessity to build long-term resilience against the associated hazards on top of improving our sub-seasonal forecasting capability.

## Introduction

The extensive peatlands on the island of Borneo in Southeast Asia are susceptible to anthropogenic intense and widespread fires which can last for months^1,2^. Many studies attribute the fires to moisture depletion in peat soils which enhances their flammability^3–6^, following a combination of rainfall deficit during the dry season in boreal summer and drainage activities^7–10^. Extremely dry conditions caused widespread fires in 1997 which burned 0.73 Mha of peatlands in Indonesian Borneo and released up to 280 megatonnes of carbon into the atmosphere, amounting to almost 5% of global fossil fuel carbon emissions that year^11^. More recently, fires over the Central Kalimantan peatlands in 2015 caused an estimated 100,000 premature deaths, major economic disruption with a cost of 16.1 billion US Dollars to the Indonesian economy, emitted more carbon in three months than the entire EU^12^, and were the primary cause of a transboundary air pollution event across Southeast Asia lasting for two months^13^.

Fire risk in Indonesian Borneo is strongly associated with the variability of dry season rainfall, with well-studied linkages including the El Niño Southern Oscillation (ENSO) and the Indian Ocean Dipole (IOD)^7,8,10,14^. During the development phase of an El Niño, the Bjerknes feedback promotes the reversal or weakening of trade winds across the equatorial Pacific upon warming of the Central-Eastern Pacific. This anomalous atmospheric circulation is associated with the subsidence arm of the Walker Circulation covering the Maritime Continent including Borneo that acts to suppress rainfall in the region^15^. The vast majority of state-of-the-art climate models realistically capture the link between ENSO and Maritime Continent rainfall^16,17^. The positive phase of the IOD cools the southern seaboard of the Western Maritime Continent^18,19^ and causes drought over Sumatra^20,21^, yet its effect on Borneo rainfall and fire risk is inconsistent and contested^17,22–24^. Despite the strong physical and observational evidence, the rarity of major fires mean that it is difficult to robustly characterise the roles of these large-scale teleconnections among other possible climatic factors using solely the observational record. A large, initialised ensemble of global climate simulations available at the UK Met Office can be applied to study low-frequency, high-impact weather and climate events and the atmospheric pathways driving them, on the basis that the simulations offer a much larger sample of events that enables a more robust assessment of the extremes^25–30^. We use the Global Seasonal forecasting version 6 (GloSea6) hindcast ensemble^31^ which provides a total of 672 fire seasons, which is many times greater than the observed climate (‘Methods’). We use regression, Bayesian and composite analysis^32^ (‘Methods’) to quantify the teleconnection link from ENSO to fire risk in Borneo and investigate whether and to what extent such influence is altered by other atmospheric factors. Bringing them together, we develop a deeper understanding of the plausible range of rainfall variability associated with fire risk and the large-scale climate drivers behind severe fire weather, which will enable enhanced future preparedness for major fires to build resilience.

## Results

### Testing the fidelity of simulated fire weather variability

The use of large initialised ensemble simulations for assessing climatic extremes is built on the basis that the model is able to reliably simulate the observed state of the climate^33^. In our case this refers to the climatology of fire weather in Borneo, represented by the simulated Canadian Fire Weather Index (FWI) and its main large-scale climate driver, namely the ENSO, during boreal summer. To evaluate the model simulations, we derive the FWI using the ERA5 reanalysis and use the NOAA Niño3.4 SST (referred to as ‘observational estimates’ below) covering the same timeframe as the model simulations (June-July-August (JJA) 1993–2016) to be evaluated against. The model simulations contain 28 ensemble members initialised at different times in May during this period (‘Methods’).

**Fig. 1:**
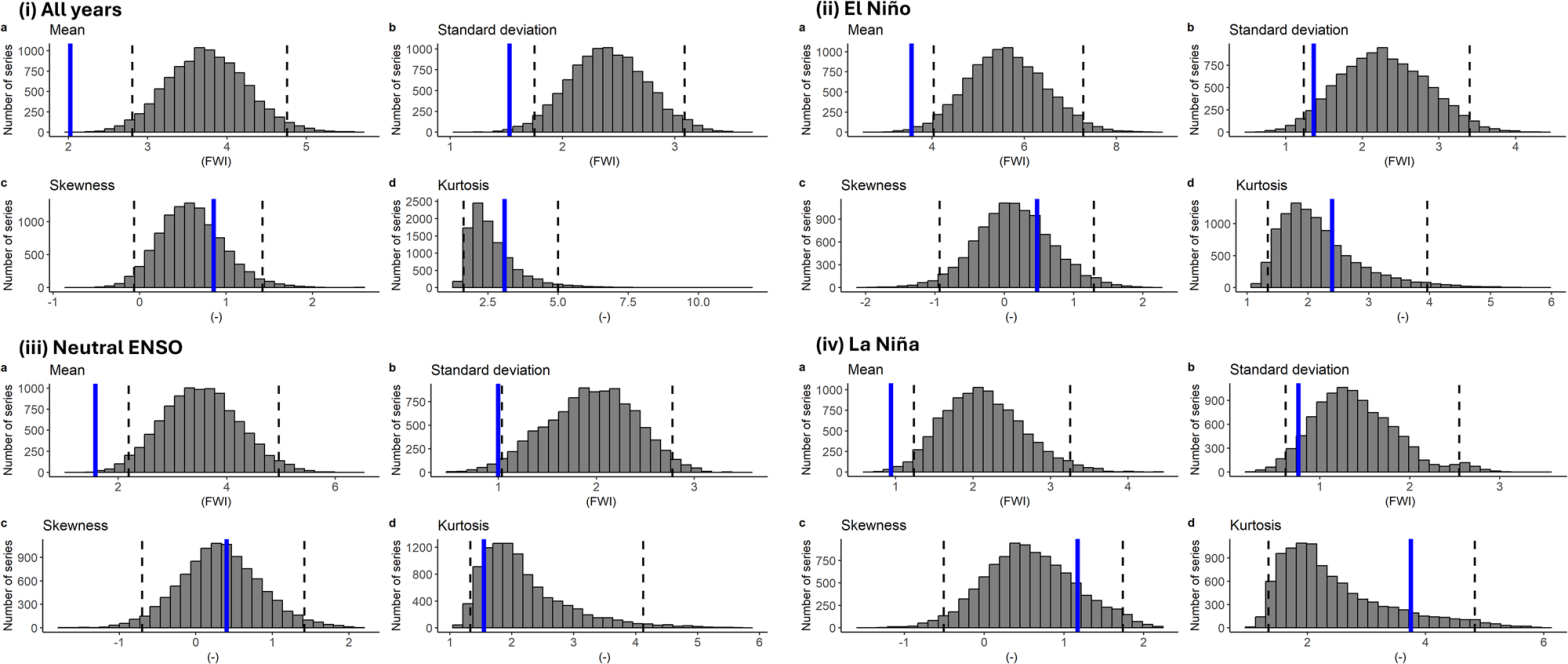
Fidelity test of GloSea6 seasonal hindcast for Borneo FWI in JJA. The blue solid line represents the observational estimate, the grey bars show the bootstrapped model (obtained by subsampling the accumulated model data 10,000 times) distributions of **a** mean, **b** standard deviation, **c** skewness and **d** kurtosis in (i) all year, (ii) El Niño only, (iii) neutral ENSO only and (iv) La Niña only, and the black dotted lines indicate the boundaries within 95% of the model distribution^76^.

**Fig. 2:**
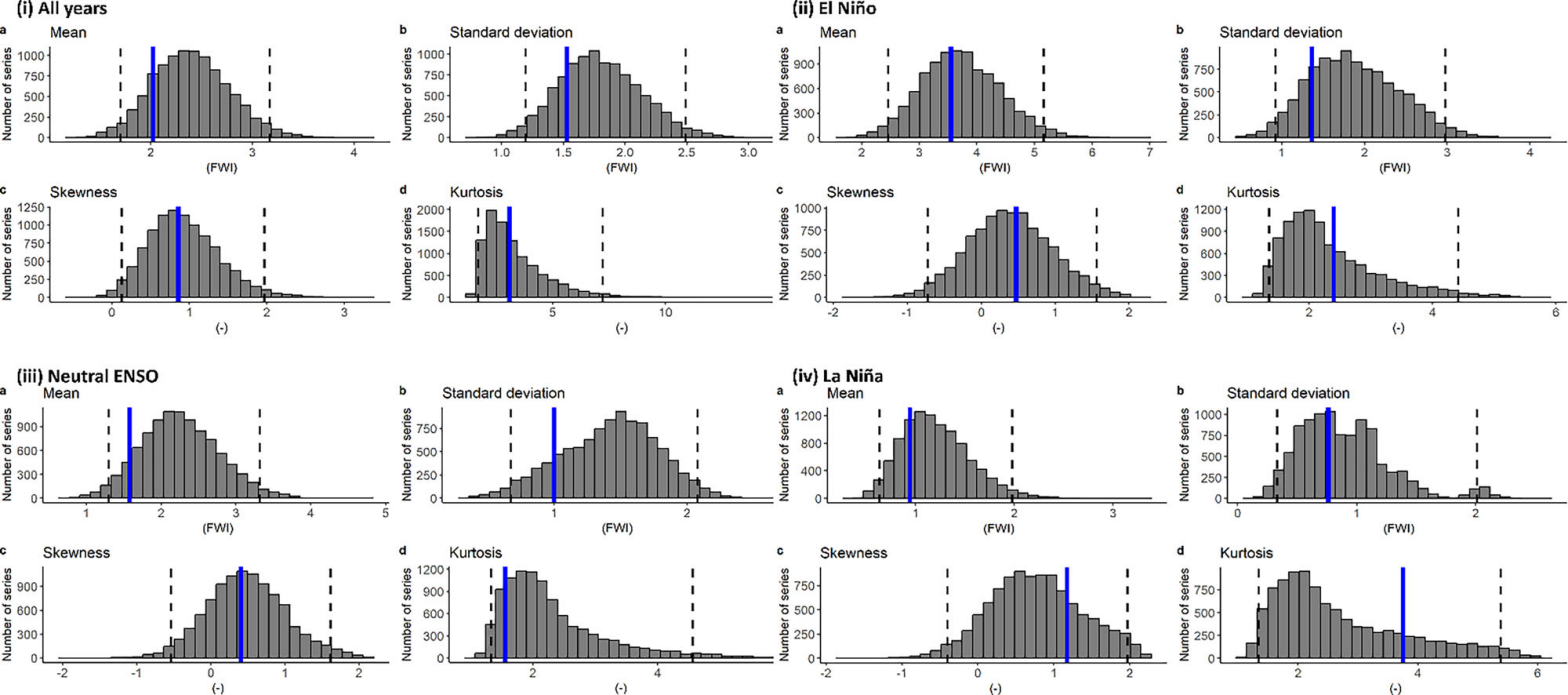
Fidelity test of GloSea6 seasonal hindcast for Borneo FWI in JJA based on bias-corrected rainfall using quantile mapping. The grey bars show the bootstrapped model distributions of **a** mean, **b** standard deviation, **c** skewness and **d** kurtosis in (i) all year, (ii) El Niño only, (iii) neutral ENSO only and (iv) La Niña only, and the black dotted lines indicate the boundaries within 95% of the model distribution^76^.

**Fig. 3:**
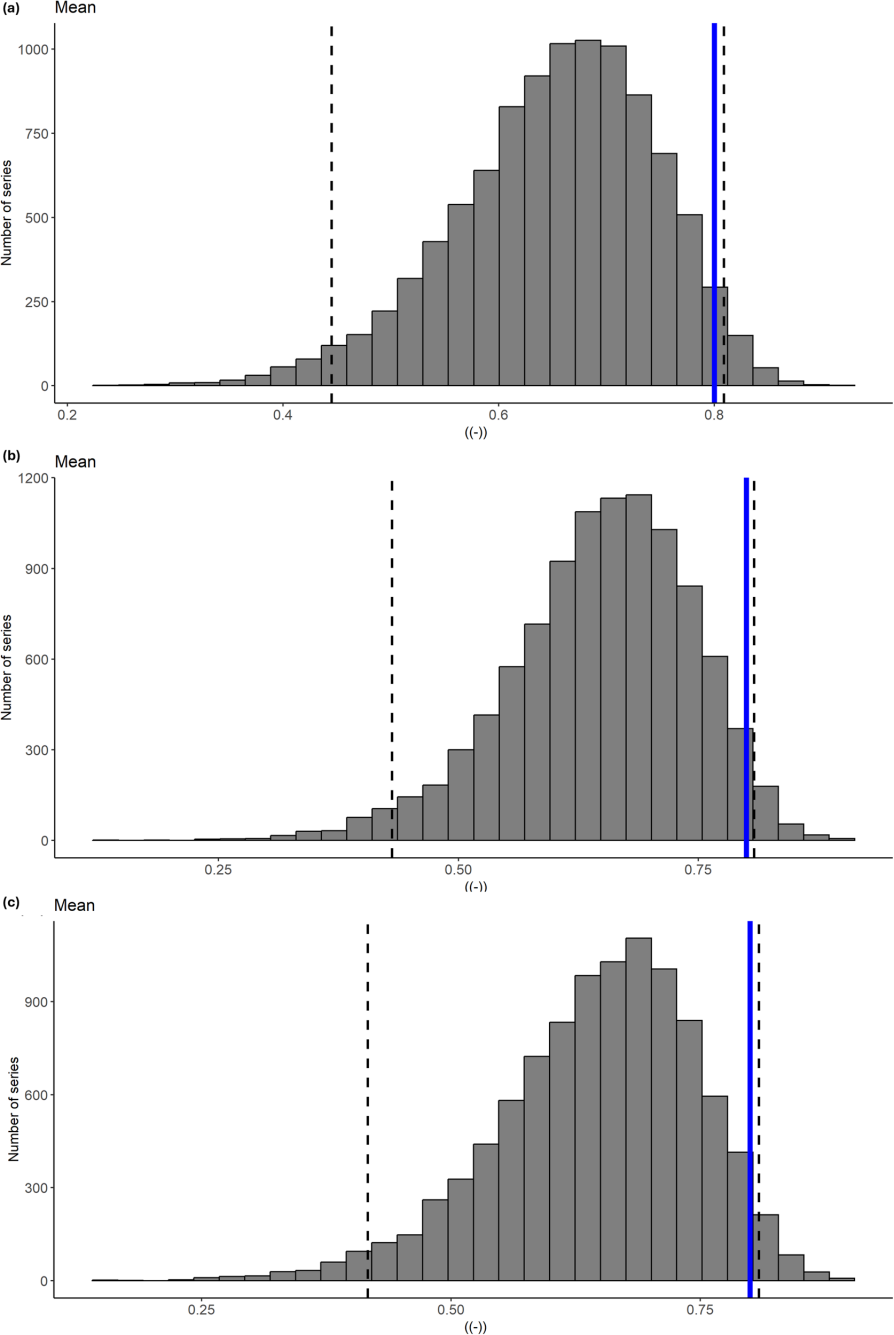
Fidelity test of GloSea6 seasonal hindcast for the correlation between ENSO and Borneo FWI in JJA. The simulated FWI are based on **a** raw GloSea6 rainfall, **b** bias-corrected rainfall using a multiplicative factor and **c** bias-corrected rainfall using quantile mapping. The blue solid line represents the observational estimate, the grey bars show the distribution of correlation coefficients between the subsampled (by 10,000 times) FWI and Niño 3.4 SST, and the black dotted lines indicate the boundaries within 95% of the model distribution.

Given the context of our study, we pay particular focus as to the model’s ability to capture the effect of ENSO, the main teleconnection pathway of teleconnection to the fire risk in Borneo. We test the fidelity of the model in two ways (‘Methods’) to examine whether it is able to realistically simulate the fire weather statistics^25^ and represent the teleconnection pathway concerned^29,32^. Overall, the model suffers from a systematic dry bias with an underestimation of rainfall by approximately 50%, resulting in an overestimation of FWI (Fig. 1(i)). Such bias is consistent across the El Niño, Neutral and La Niña phases of ENSO (Fig. 1(ii)–(iv)), which implies that ENSO representation is not the source of the rainfall bias, echoing with other studies that attribute this to the energy flux representations across the tropics and subtropics^34–36^. Upon bias-correcting the simulated rainfall data using quantile mapping or a multiplicative factor with reference to the observational estimates (‘Methods’), the observed FWI falls within the 95% confidence interval of the model distribution, under all ENSO phases (Fig. 2, Fig. S1). The observational estimate for the correlation coefficient between the FWI and Niño3.4 lies within the central 95% of the model correlation regardless of whether bias correction is applied (Fig. 3), despite the fact that the observed correlation coefficient is slightly greater than the ensemble mean. To summarise, our fidelity test provides robust evidence that the model ensemble is able to sufficiently capture the teleconnection link from ENSO to Borneo fire risk, enabling the analysis of the relative risk of major fires conditioned on the large-scale climate driver.

### Borneo FWI vs. ENSO in the simulations and observations

**Fig. 4:**
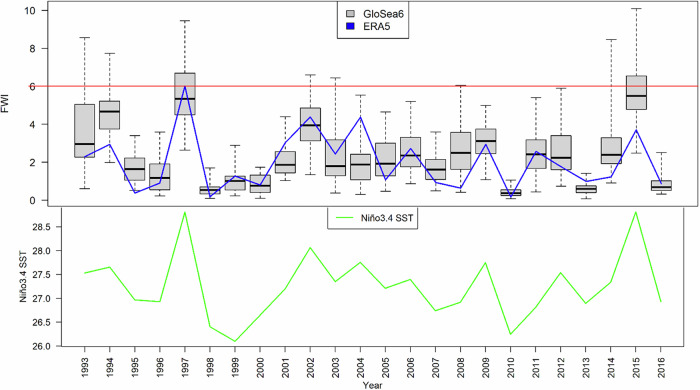
Borneo FWI in JJA simulated by GloSea6 hindcast ensemble based on bias-corrected rainfall by quantile mapping (in grey/black boxes and whiskers) and estimated by ERA5 (in blue line). For each year, the whisker represents the ensemble spread, the box indicates its interquartile range and the black line within it shows the ensemble median. The lower panel shows the corresponding Niño3.4 SST estimated by NOAA (in green line). The red horizontal line corresponds to the observed maximum.

In the observational estimates, years of high FWI always coincide with an El Niño (high Niño3.4 SST) (Fig. 4). The model simulations (the boxes and whiskers) also reflect this association, with the FWI medians and ranges being much higher when an El Niño is taking place. However, the model provides a strikingly larger range of FWI under each ENSO phase. Even though strong El Niño occurred only twice in the last 24 years (1997 and 2015), 11 out of 24 years saw at least one ensemble member falling above the ensemble median of the two years, implying the existence of major fire risk in these many years. To show a more realistic estimate, the FWI in Fig. 4 is based on bias-corrected rainfall using quantile mapping, yet our conclusion on the model range under each ENSO phase holds regardless of whether bias-correction is applied (Fig. S2).

**Fig. 5:**
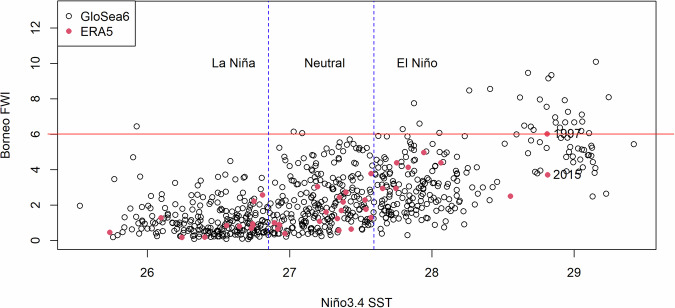
Borneo FWI vs. Niño3.4 SST in JJA, simulated by GloSea6 hindcast ensemble based on bias-corrected rainfall by quantile mapping (black) (1993–2016), and estimated by ERA5 (FWI) and NOAA (Niño3.4 SST) (pink) (1981 – 2019). The red horizontal line corresponds to the observed maximum, whereas the blue vertical dotted lines refer to the stratification of ENSO phases.

The correlation between Niño3.4 SST and FWI is strongly positive with a beta coefficient of greater than +0.7 (Fig. 4), also shown by in the scatter plot of Borneo FWI versus Niño3.4 SST in the model (1993–2016) and observation (1981–2019) (Fig. 5). Figure 5 also reflects the plausible range of FWI under each ENSO phase. Although ENSO clearly modulates fire risk and leads to the worst possible events with many of the highest values in the El Niño category, there are a few simulated fire seasons with FWI greater than the observed maximum in 1997 under a much lower Niño3.4 SST. This shows that the threat of major fires in Borneo exists without an El Niño, even during a La Niña when above average rainfall is expected.

### Risk of major fires conditioned on climate driver

**Fig. 6:**
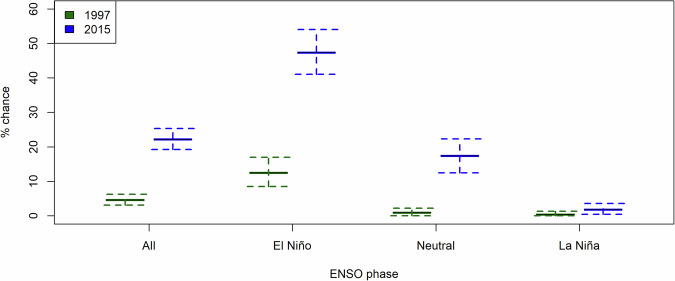
Chance of FWI exceeding 1997 and 2015, conditioned on ENSO phases, simulated by GloSea6 hindcast ensemble based on bias-corrected rainfall by quantile mapping. The central mean estimate (solid lines) is based on the number of runs with FWI exceeding these values divided by the total number of runs, while the error bars (whiskers) are constructed by bootstrap resampling for 10,000 times for each ENSO phase.

The model simulations show that an El Niño substantially enhances fire weather and elevates the risk of major fires in Borneo (Fig. 6). Considering the simulated FWI based on bias-corrected rainfall and the observed maximum FWI as a benchmark of ‘major fires’, an El Niño raises the chance of major fires by a factor of 2.7 (‘Methods’). The risk of major fires is not negligible under ENSO-neutral or even La Niña. When considering 2015, another year when damaging fires occurred, as a benchmark (FWI = 3.70), the rates of exceedance are 17.4% and 1.8% under ENSO-neutral and La Niña respectively. This warrants further investigation of the drivers behind these unprecedented simulated events, especially given that fires at the scale of 1997 and 2015 have never been known to occur without an El Niño.

### Dynamics influencing the range of FWI given an ENSO phase

The GloSea6 model ensemble is skilful in representing the ENSO teleconnection pattern in relation to the fire risk. This means the range of outcomes under different ENSO phases may be considered physically plausible, despite a lack of observational precedents due to the short record. Here we analyse the underlying drivers of the simulated events of interest, particularly where the simulated fire risk is major under neutral or La Niña conditions.

**Fig. 7:**
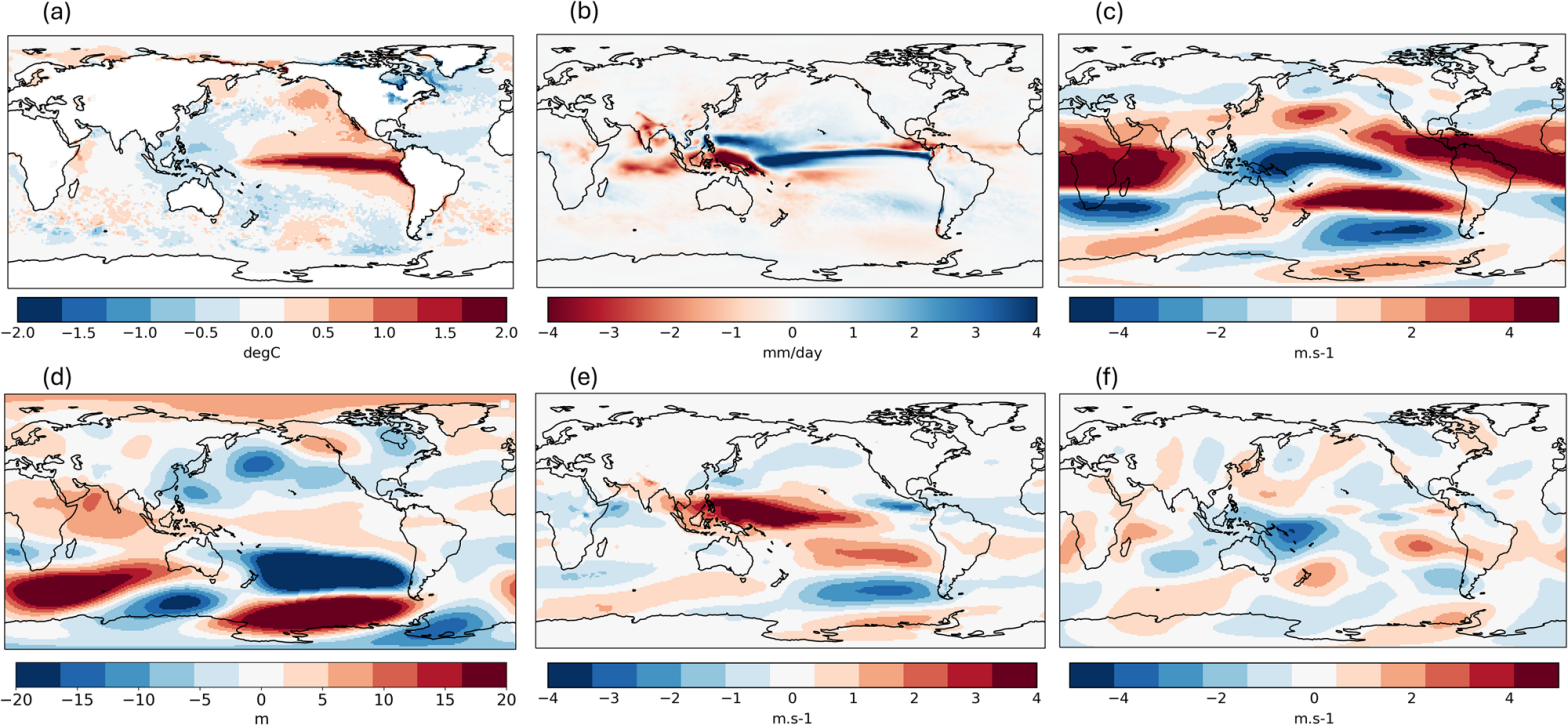
El Niño minus La Niña composites. The maps show JJA global **a** SST **b** precipitation **c** zonal wind at 200 hPa (positive value indicates westerly wind) **d** geopotential height at 500 hPa **e** zonal wind at 850 hPa **f** meridional wind at 200 hPa of GloSea6 seasonal hindcast runs comparing between El Niño (*n* = 224) and La Niña (*n* = 224) ENSO phases.

**Fig. 8:**
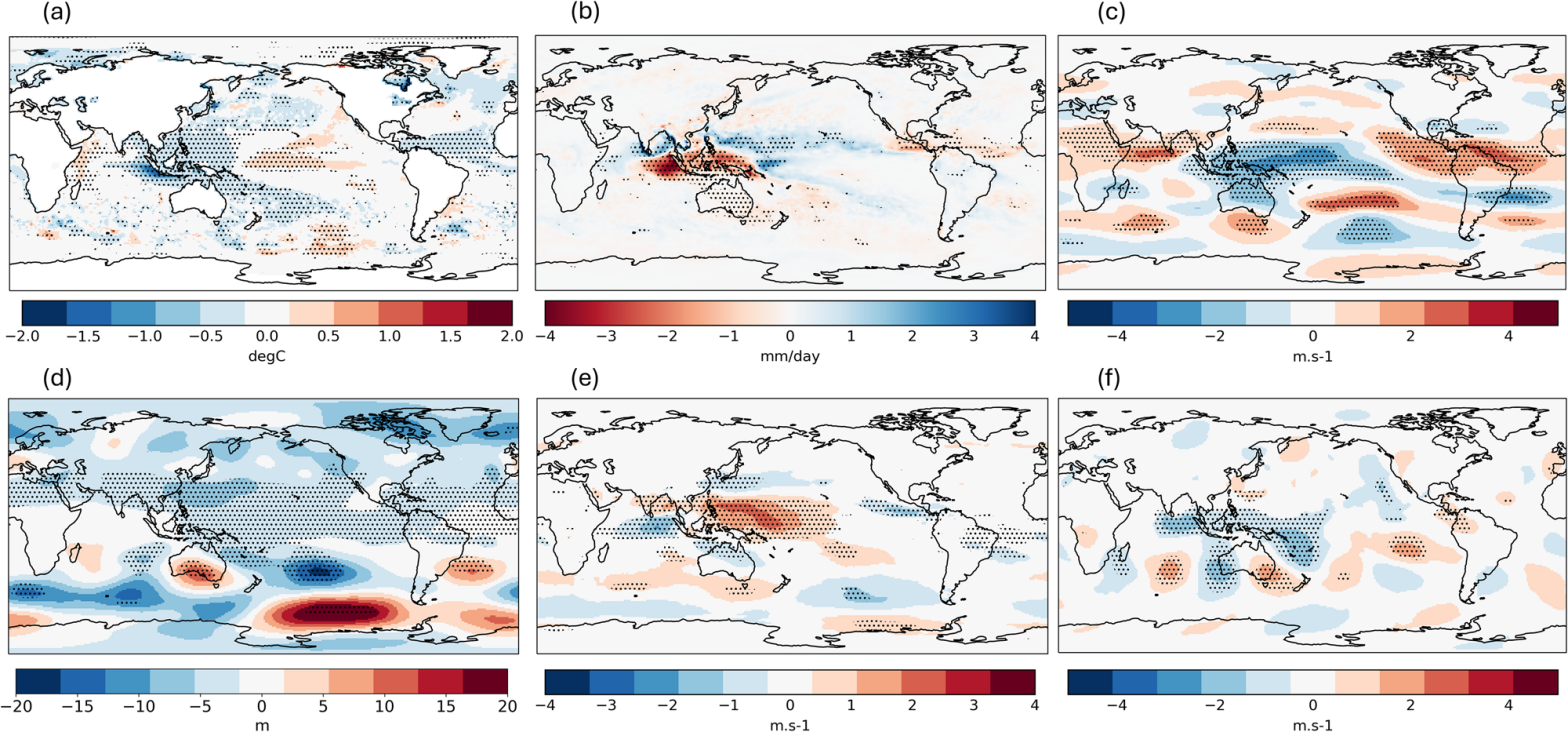
Global circulation patterns during non-El Niño high FWI seasons. The maps show composite of anomalies of JJA global **a** SST **b** precipitation **c** zonal wind at 200 hPa **d** geopotential height at 500 hPa **e** zonal wind at 850 hPa **f** meridional wind at 200 hPa of GloSea6 seasonal hindcast runs simulating FWI above 2015 (3.70) under neutral or La Niña ENSO phases (*n* = 43). Stippling indicates significant difference from zero at 95% confidence level based on a two-sided Student’s *t* test (corrected by Benjamini-Hochberg false discovery rate), by comparing between the simulated runs and the ensemble mean under the respective ENSO phase.

The primary cause of a low rainfall anomaly over the Maritime Continent is anomalous subsidence over the region inhibiting convective activity, which an El Niño acts to remotely force^15^. The question is thus: what are the other factors that influence the strength of this subsidence? To answer this, we first identify the most relevant atmospheric features associated with the El Niño-driven subsidence by plotting the composite of simulated global circulation patterns in comparison between the El Niño and La Niña phases of ENSO (Fig. 7), which agrees well with the observed patterns (Fig. S3). The main feature is the westerly wind anomaly in the lower troposphere reflecting the weakening or reversal of trade winds over the equatorial Western Pacific (Fig. 7e) and the easterly wind anomaly in the upper troposphere over the same region (Fig. 7c) corresponding to the drought-causing (Fig. 7b) anomalous subsidence arm of the Walker circulation. The same atmospheric features exist during events in which the simulated Borneo FWI exceeds that observed in 2015 without an El Niño (abbreviated as ‘simulated non-EN major events’ below; Fig. 8b, c, e). In these cases, the Pacific trade winds mimic an El Niño atmospheric response, but in the absence of a large-scale SST mode.

**Fig. 9:**
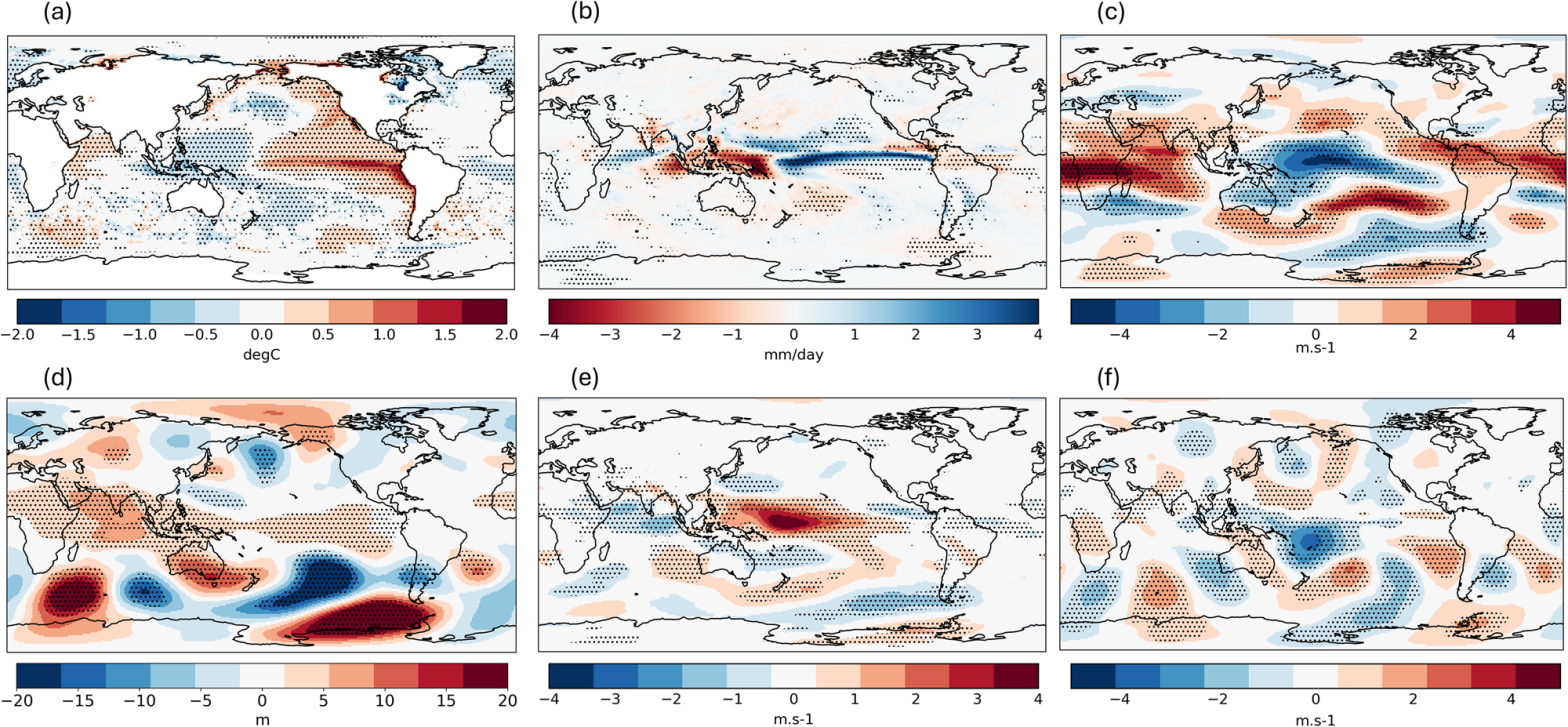
A comparison of global circulation patterns during El Niño events (*n* = 224) with high vs. low FWI. The maps show the differences between higher (*n* = 112) and lower (*n* = 112) than the median FWI given an El Niño (3.62), based on JJA global **a** SST **b** precipitation **c** zonal wind at 200 **d** geopotential height at 500 hPa **e** zonal wind at 850 hPa **f** meridional wind at 200 hPa simulated by GloSea6 seasonal hindcast. Stippling indicates significant difference from zero at 95% confidence level based on a two-sided Student’s *t* test (corrected by Benjamini-Hochberg false discovery rate).

Other than the Bjerknes feedback within the equatorial Walker Circulation as the main driver, equatorward propagation of a Rossby wave train from the southern extratropics have previously been shown to affect the Pacific trade winds during boreal summer and contributed to the development of super El Niño events^37^. We find a similar wave pattern as shown in the mid-troposphere geopotential height and upper-troposphere meridional wind anomalies during the simulated non-EN major events, which stretches through the mid-latitude South Indian Ocean into the South Pacific (Fig. 8d, f). The high-pressure ridge over Southern Australia and the mirroring low pressure over the South Pacific are consistent with a low-level southeasterly wind anomaly over Northeastern Australia (Fig. 8c, e). The Rossby wave then eventually turns into the equatorial Pacific acting to reverse the trade winds, consistent with previous work^37^. The extratropical-tropical nature of this teleconnection pathway is also evidenced by anomalous northward Takaya-Nakamura wave activity flux^38^ over the west of the study region, with the climatological poleward flux weakened in the ensemble members during simulated non-EN major events, to nearly net zero in the only ensemble member where FWI exceeded the observed value in 1997 during a La Niña (Fig. S4). This extratropical forcing is also evident in driving the spread in rainfall and fire risk response in Borneo to an El Niño in the model (Fig. 9b–f) and may have contributed to suppressing the fire risk in 2023 despite a developing El Niño (Fig. S5).

The rainfall anomaly composite of simulated non-EN major events shows the largest dry anomaly towards the western end of Maritime Continent, over Sumatra and its southern seaboard (Fig. 8b). This resembles the rainfall pattern of a positive IOD event^21^. The SST anomaly pattern over the Indian Ocean also signals the onset of positive IOD^19^, showing the strongest negative anomaly over the Sumatran and Javan coast (Fig. 8a). The mean Dipole Mode Index (DMI) under these events (*n* = 43) is +1.27, signifying a moderately positive IOD on average. The positive IOD lowers the SST across the western Maritime Continent region (Fig. 8a) and suppresses rainfall in Borneo through local air-sea interaction^15^. Furthermore, the enhanced uplifting over the Western Indian Ocean during the positive IOD may strengthen the regional branch of Hadley cell, cause an equatorward shift of westerly jet in the eastern Indian Ocean, and may augment the aforementioned equatorward flux^39^. Notwithstanding the weak association between the IOD and Borneo rainfall based on the observational data^17,23^, these simulated events imply an elevated chance of major fires in Borneo under a positive IOD in the absence of an El Niño. We note a relatively weak link between IOD and Borneo rainfall (and a stronger link with the ENSO) during the study period compared to that in the mid-20^th^ century^17^. Meanwhile, the observed IOD-Borneo FWI correlation is slightly weaker than that simulated by the model (Fig. S6) while the observed ENSO-Borneo FWI correlation is slightly stronger than the simulated link (Fig. 3) (with both links falling within the central 95% of the model distribution), which may reflect such multidecadal variability. The ongoing weakening trend of ENSO-IOD relationship^40^ may raise the possibility of major fires in Borneo under lower Niño3.4 SSTs over the next few decades. Therefore, we advocate future work to disentangle the relative roles of IOD, local SSTs and extratropical drivers in influencing this unprecedented combination.

## Discussion

Even over tropical regions strongly influenced by large-scale ocean-atmosphere coupled modes (namely the ENSO and IOD), there have been cases where rainfall did not respond accordingly. As an example, rainfall in Northern Northeast Brazil was above normal during an El Niño in 2019 against the expected drying response^41^. Yet, studies tackling this issue are extremely scarce. We challenge the well-established notion that Borneo fires are ‘El Niño fires’, as was the case in the observational record. Analysing large ensemble data from GloSea6, we have quantified the influence of ENSO and discovered alternative teleconnection pathways leading to major fire risk in Borneo. We find, in common with studies conducted over other tropical regions, that equatorward-propagating Rossby waves from the mid-latitude winter hemisphere (for instance, the Southern Hemisphere during JJA) can play a modulating role on the rainfall response to the large-scale coupled mode^29,41^. Although such extratropical influences are shown to only affect rainfall at intraseasonal timescale in the observations, our study shows that under extraordinary circumstances, they may contribute to the decoupling of atmospheric responses from the ocean mode, leading to significant anomaly signatures spanning over boreal summer. Our precipitation anomaly composites show that this decoupling effect is not confined to our study region but may even alter the geographical distribution of tropical rainfall belts at global scale (Fig. 8b). This may have implications on the other parts of the globe as well, as tropical-extratropical teleconnections are primarily triggered by the huge amounts of energy released from the intense tropical rainfall serving to alter the Rossby wave sources^42^, with the spatial distribution of rainfall having a profound effect on the teleconnection pathways^43^. Therefore, further studies on the characterisation, drivers and monitoring of the decoupling phenomenon will offer better predictability of ENSO-related high-impact weather across the globe.

In view of the devastation caused by low frequency, high-impact weather events and to assess their impacts, many studies have used large forecast ensembles with a climatic application to find the range of variability which may be outside the historical precedents yet deemed physically plausible^25,33,44–48^. The readily available ensemble outputs provide a set of realisations much larger than the observations, enabling risk assessment of climatic extremes more robustly^25,49^. If the model ensemble is proven to be able to simulate the observed climate in the region of interest to a reasonable degree, the model may be used for evaluating the probability of unprecedented extremes under the present climate, known as the UNprecedented Simulated Extremes using ENsembles (UNSEEN) method^25^. We address the ‘unprecedented’ nature of an event not only with reference to its rarity but also with respect to the large-scale atmospheric driver. This is because the level of preparedness against fires is enhanced under the anticipation of an El Niño^50^, such that much greater impacts might be expected in case of major fires without an El Niño. In our case, due to the severe dry bias in the model simulation (Fig. 1), we refrain from interpreting the percentage of ensemble members falling beyond the observed record as the chance of an unprecedented fire season. Despite this, given the model’s proven ability to capture the teleconnection pathway, it can still provide insights regarding the atmospheric modes leading to rainfall variability, and in particular the unprecedented combination of severe fire risk under a cool Niño3.4 SST. In fact, climate forecasting systems tend to capture the interannual variability of tropical rainfall and its connection to large-scale climate modes much better than its mean climatology^51^. We therefore advocate causality-based studies using large initialised ensembles to be carried out for climate risk assessment purpose over regions where more than one predictable climate drivers are influential. Depending on the level of knowledge of causal drivers, we may decide between causal methods including knowledge-guided causal inference^32^ or more data-driven, causal discovery approaches^52–56^ for analysing the ensemble data to quantify and uncover the unprecedented yet physically plausible drivers to high-impact events.

Our approach for evaluating teleconnections to major fires in Borneo aims not only to assess risk but also for informing their predictability in a transparent and traceable manner^32^. Our study region is characterised by the predictability of dry season rainfall at seasonal timescale being among the highest across the globe, thanks to its strong association with ENSO, also a highly predictable large-scale climate mode^51,57,58^. Several studies harnessed this source of predictability and developed sophisticated fire forecasting systems in our study region^7^, which can be based on seasonal forecast outputs^10^. By enlarging the event set, our data-informed approach can be combined with these forecasting tools to further enhance the robustness of prediction, particularly regarding the chance of high-end, unprecedented events which is not typically communicated in operational seasonal forecasts^58^. On the other hand, given the plausible range of outcomes under a given ENSO phase, we stress that the issue of predictability limit must be realised and communicated to decision-makers^59,60^. Ultimately, long-term measures with a focus on enhancing the resilience of peatlands to droughts, including rewetting^61^, reforestation^1^ and promoting sustainable forms of livelihood^62^, are needed as prevention strategies against major fires in each and every year.

## Methods

### Study area

We defined a region over the central Maritime Continent (bounded by 2.76° S–3.21° N, 109.81–118° E)^63^ to represent the region of the Island of Borneo on which to focus. The climatology of the Indonesian Borneo region is divided into two distinct seasons, namely the wet season (November–May the following year) and the dry season (June–October), related to the seasonal shift of the Intertropical Convergence Zone (ITCZ). The dry season is caused by the northward shift of the ITCZ away from the Maritime Continent amid the development of the Asian Summer Monsoon^64^. Average monthly rainfall for the region during the dry season is around 180 mm^63^. Previous studies show high spatial coherence of interannual variability of dry season rainfall across the study region^17,65^.

### Data

For the observational data, we used a total of 24 years (1993–2016) of daily ERA5 Fire Weather Index (FWI) data in JJA, the fire season in Borneo considered in previous work^17,66^, from Copernicus Emergency Management Service^67^. We note that this data is based on ERA5 rainfall which may be prone to model errors, thus have examined other datasets to confirm that the rainfall input is reliable and conforms the satellite and in-situ measurements^17^. We used Niño 3.4 data from NOAA as an indicator of ENSO during the same time period.

For model data, we used the hindcast ensemble of Met Office Global Seasonal forecasting version 6 (GloSea6)^31^. With HadGEM3-GC3.2 serving as the base model^68^, the seasonal forecast model is of the latest generation, initialised with the atmosphere, ocean, land and sea-ice from reanalysis data, and uses the stochastic kinetic energy backscatter scheme version 2 (SKEB2) as a perturbed parameterisation scheme to generate an ensemble of simulations^69^. Daily model data at a horizontal resolution of approximately 60 km were extracted. Using the available hindcast simulations initialised every May (the 1st, 8th, 15th and 22nd, with seven ensemble members each day) from 1993 to 2016 (a total of 24 years) before the targeted JJA, the GloSea6 provides 28 realisations of FWI simulations and their respective associations with ENSO (represented by Niño 3.4 SST) per year, serving to sufficiently enlarge the event set to a total of 672 fire seasons, or 28 times greater than the observed.

### Fire Weather Index

We used the Canadian Fire Weather Index (FWI) to estimate fire risk in the study region. The FWI was developed by Canadian Fire Weather Service based on precipitation, surface air temperature, wind speed and relative humidity. Based on these inputs, the moisture content in organic matter is evaluated, represented by Drought Code, Duff Moisture Code and Fine Fuel Moisture Code. These give immediate indices of FWI, namely Buildup Index and Initial Spread Index, which reflects the potential of fire onset and possible speed of fire spread respectively. Details of the index can be found in its documentation^70^.

Despite being originally developed in Canada, the FWI has been used extensively across the globe to evaluate fire risk, including in the Asian tropics^8,71^. The index closely resembles fire occurrence in the Maritime Continent region, based on validation using satellite data from the Global Fire Emissions Database^72^ and the World Along Track Scanning Radiometer fire atlas^73^. This is because fire occurrence in the region is strongly related to rainfall deficit, a major component of FWI computation^72^. With its proven ability, the FWI has been used both for early warning^73,74^ and climatic studies^66^ in Borneo.

We computed the daily FWI using the meteorological inputs from GloSea6 on its native grid and obtained the area average within the study region. The values for each day were then averaged to obtain a JJA mean for each year to generate a time series which reflects the interannual variability.

### Model fidelity

We employed fidelity tests to examine whether the model realistically reproduces the observed state of climate. To do this, 10,000 sets of proxy time series were generated by bootstrap resampling^75^ of the model data accumulating all the available years, initialisation dates and ensemble members. If the statistical measures (see next paragraphs) of the observational estimate fall within the central 95% of the bootstrapped model distribution, it can be argued that the observation is statistically indistinguishable from the model^25,33,76^, in other words, the model can be regarded as being able to realistically simulate the fire weather statistics^25^ and represent the causal pathway studied^29,32^.

We conducted the fidelity test to determine whether the teleconnection pathway from ENSO to fire risk in the study region is realistically represented in the GloSea6 ensemble. The fidelity test was achieved by two methods: (a) evaluating the FWI model distributions under each of the ENSO phases (El Niño, Neutral and La Niña, stratified into terciles based on the Niño3.4 climatology^32^), and (b) evaluating the model distribution of correlation coefficients between the FWI and ENSO (Niño3.4 SST). For (a), the mean, standard deviation, skewness and kurtosis of each proxy time series were calculated and compared against the observational estimate. For (b), we fitted each pair of the standardised proxy time series (*FWI* and *ENSO*) into the following equation:

1 $${FWI}={x}_{ENSO- > {FWI}}(ENSO)+c,$$

where *x*_*ENSO->FWI*_ is the beta coefficient and *c* represents noise. Backed by our physical knowledge that ENSO exerts influence on fire risk^14^, each beta coefficient (a total of 10,000) reflects the associated physical mechanism in each resampled ensemble run^32^. We consider that this teleconnection pathway is satisfactorily reproduced by the model if the beta coefficient based on the observational estimates falls within the central 95% of the model distribution^29^.

### Effect of ENSO on fire risk

We use a Bayesian approach^32^ to estimate the effect of ENSO on fire risk in the study region. Such effect is expressed as a Bayes Factor using the following equation:

2 $$BF\left(FWI+,\,ENSO+\right)=P(FWI+|ENSO+)/P\left(FWI+\right),$$

where *BF* (*FWI* + *, ENSO* + ) represents the increase in probability of FWI above a given threshold under the presence of El Niño (*ENSO* + ). ENSO is stratified into terciles describing El Niño, neutral and La Niña conditions, whereas FWI is stratified into two categories, FWI+ and FWI-, representing above and below the threshold respectively. *P* (*FWI* + ) is the percentage of model simulations falling above the threshold (regardless of the ENSO state), whereas *P* (*FWI* + | *ENSO* + ) is the percentage of those falling above the threshold *given that* an El Niño is simulated.

In our case, considering the simulated FWI based on bias-corrected rainfall (Figs. 5), 4.6% of all simulated fire seasons exceed the observed maximum in 1997 (FWI = 6.01) (*P (FWI* + ) = *0.046*). This increases to 12.5% under an El Niño (*P (FWI* + | *ENSO* + ) = *0.125*). Using the observed maximum FWI as a threshold of ‘major fires’, an El Niño raises the chance of major fires by a factor of 2.7.

## Supplementary information

Supplementary Information

## Data Availability

ERA5 reanalysis data and GloSea6 seasonal hindcast data used in this study are openly available from the Climate Data Store (CDS; https://cds.climate.copernicus.eu) supported by the European Centre for Medium-Range Weather Forecasts (ECMWF) Copernicus Climate Change Service (C3S). Observed Niño 3.4 SST data was downloaded from the National Oceanic and Atmospheric Association (NOAA) Physical Sciences Laboratory website (https://psl.noaa.gov/data/timeseries/monthly/NINO34/).

## Code availability

The computer codes used for conducting this analysis and producing the figures are available from the corresponding author upon request.
