# Supplementary material for: Major fires in Indonesian Borneo are possible under all ENSO phases
Source: NPJ Nat Hazards. 2026 Apr 23;3(1):46. doi: 10.1038/s44304-026-00209-4 (PMC13106036; doi:10.1038/s44304-026-00209-4)
Supplement: Supplementary file 1 — Supplementary Information [file 44304_2026_209_MOESM1_ESM.docx]

**Major Fires in Indonesian Borneo are possible under all ENSO phases**

Timothy Lam*^1,2^, Jennifer L. Catto^3^, Gillian Kay^4^, Nick Dunstone^4^, Rosa Barciela^4^, Anna B. Harper^3,5,^

^1^Centre for Doctoral Training in Environmental Intelligence, University of Exeter, Exeter, UK 
^2^WCRP Regional Information for Society (RIfS), Montreal, Canada

^3^Faculty of Environment, Science, and the Economy, University of Exeter, Exeter, UK

^4^Met Office, Exeter, UK

^5^Department of Geography, University of Georgia, Athens, Georgia, USA

**Supplementary File**


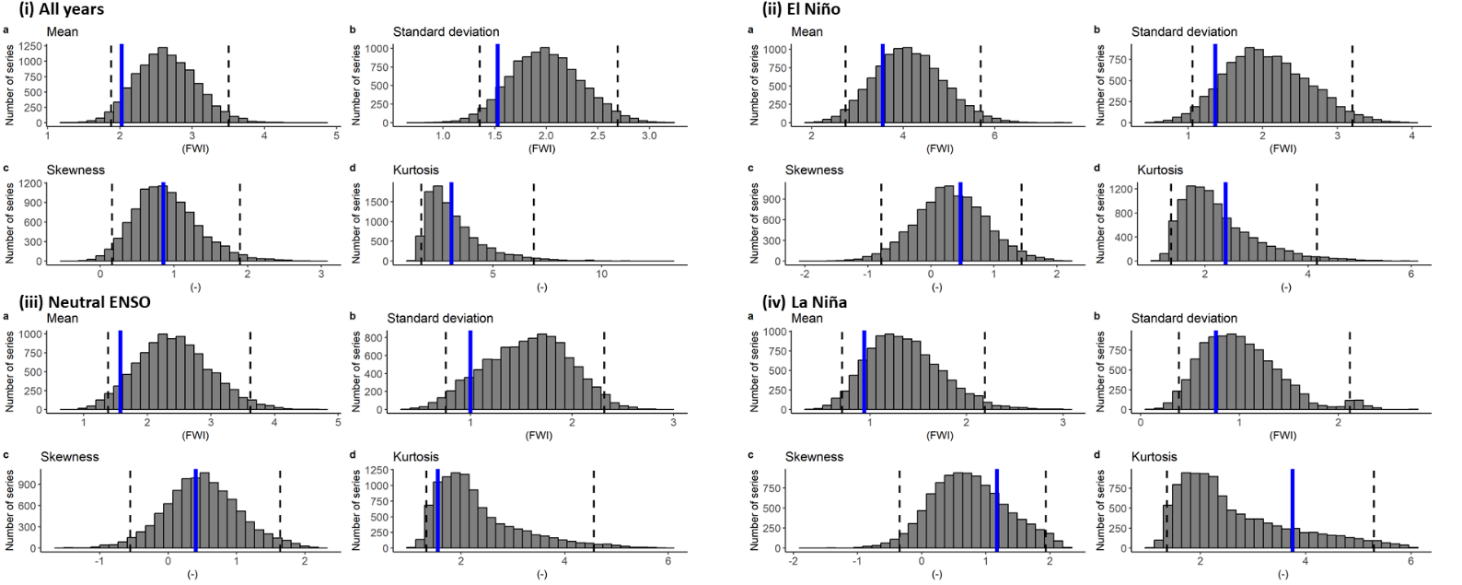


Fig. S1: Fidelity test of GloSea6 seasonal hindcast for Borneo FWI in JJA, similar to Fig. 1 but based on bias-corrected rainfall using a multiplicative factor, with simulations of (i) all year, (ii) El Niño only, (iii) neutral ENSO only and (iv) La Niña only. The grey bars show the bootstrapped model distributions of (a) mean, (b) standard deviation, (c) skewness and (d) kurtosis.


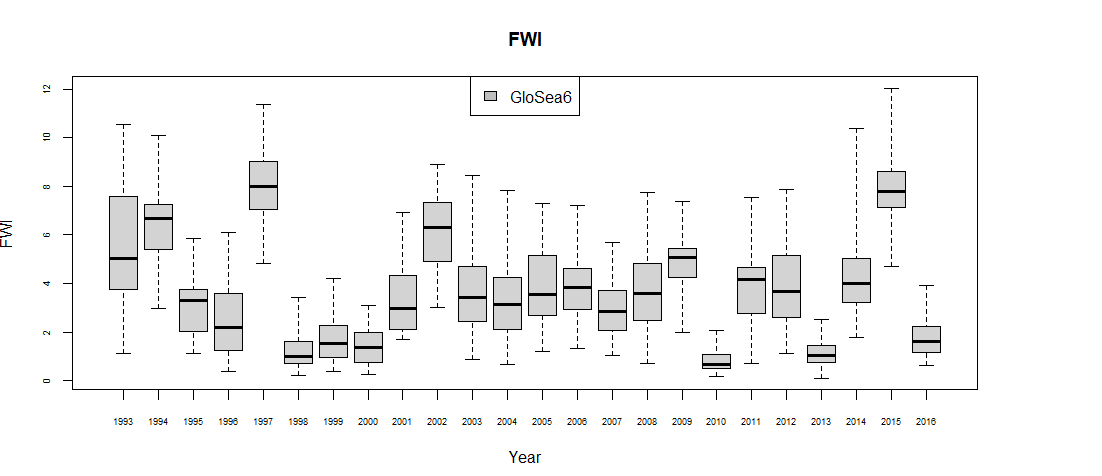


Fig. S2: Borneo FWI in JJA simulated by GloSea6 hindcast ensemble without bias correction (in grey/black boxes and whiskers). For each year, the whisker represents the ensemble spread, the box indicates its interquartile range and the black line within it shows the ensemble median


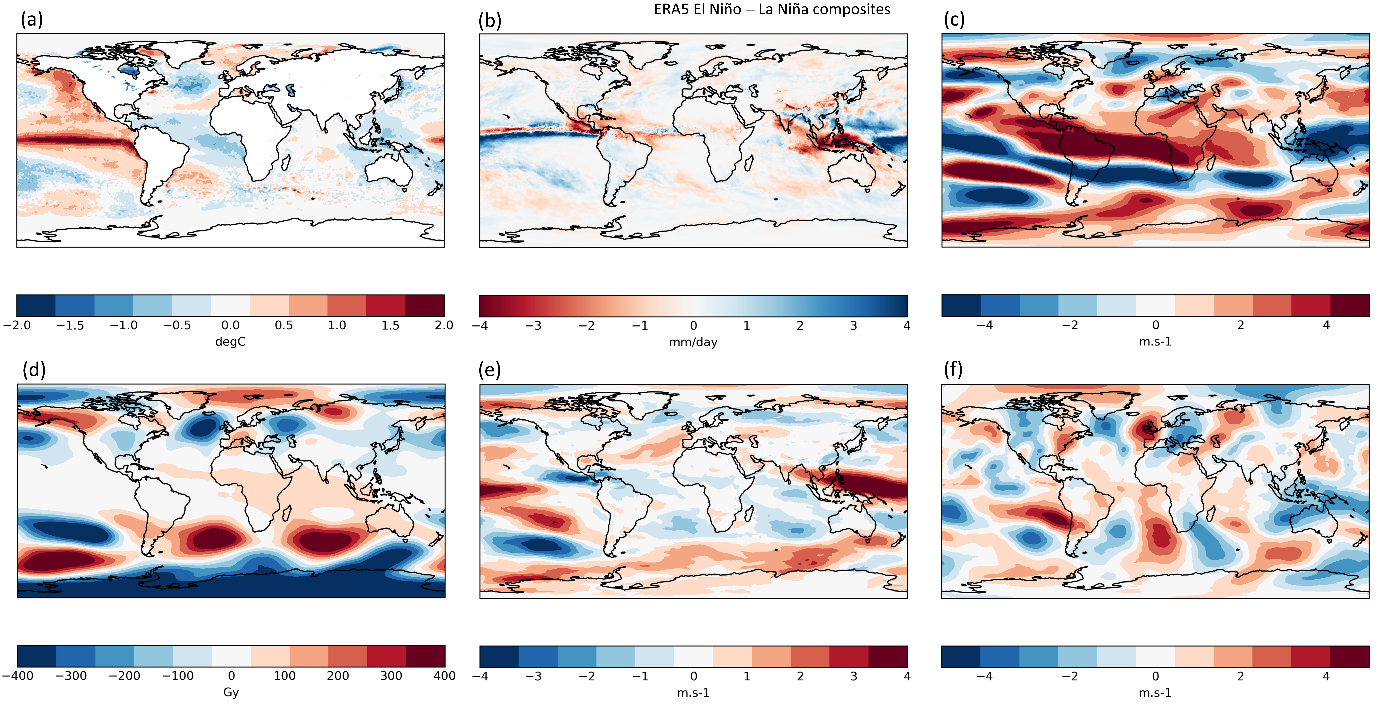


Fig. S3: Composite of anomalies of JJA global (a) SST (b) precipitation (c) zonal wind at 200 (d) geopotential height at 500 hPa (e) zonal wind at 850 hPa (f) meridional wind at 200 hPa comparing between El Niño (n = 8) and La Niña (n = 8) ENSO phases in 1993 – 2016, based on ERA5 reanalysis.


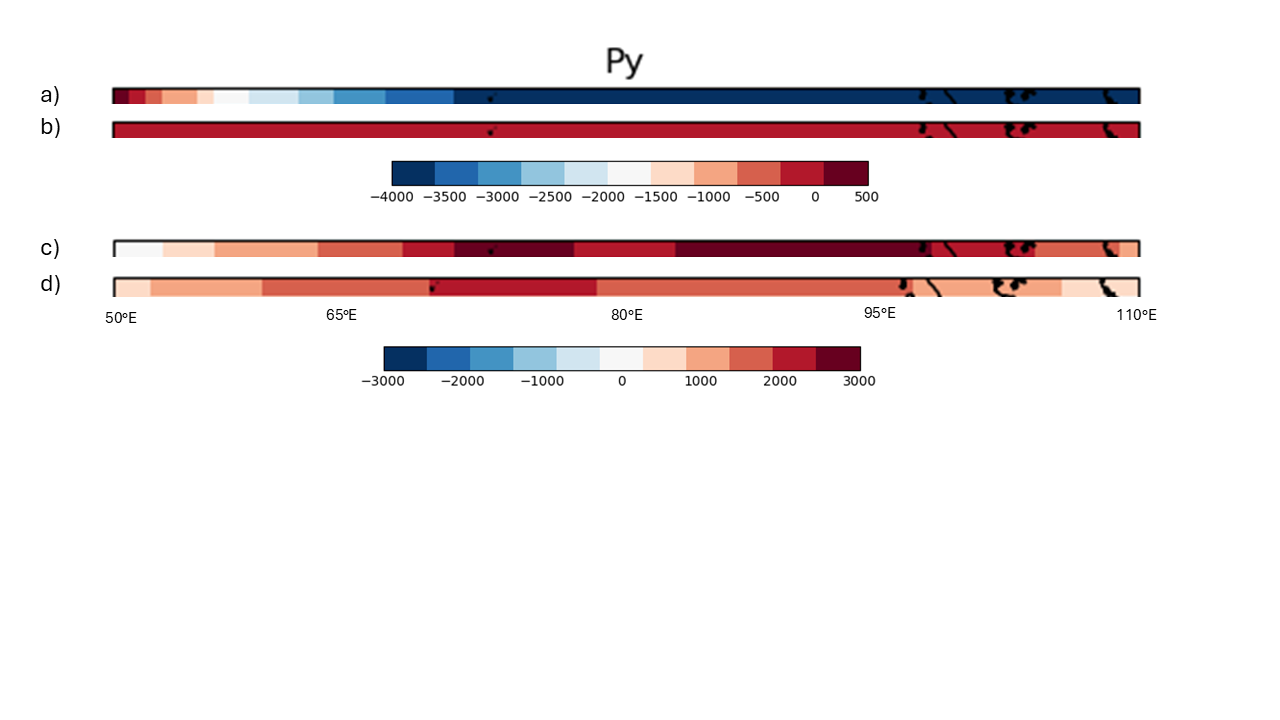

Fig. S4: Accumulation of meridional TN-WAF across 0 – 40°S over each longitudinal band in GloSea6 hindcast ensemble, for (a) climatological mean, absolute; (b) the case where simulated FWI was greater than that observed in 1997 during a La Niña, absolute; (c) the cases where FWI was greater than that observed in 1997 during La Niña or neutral ENSO, anomaly; (d) the cases where FWI was greater than that observed in 1997 during La Niña or neutral ENSO, anomaly.


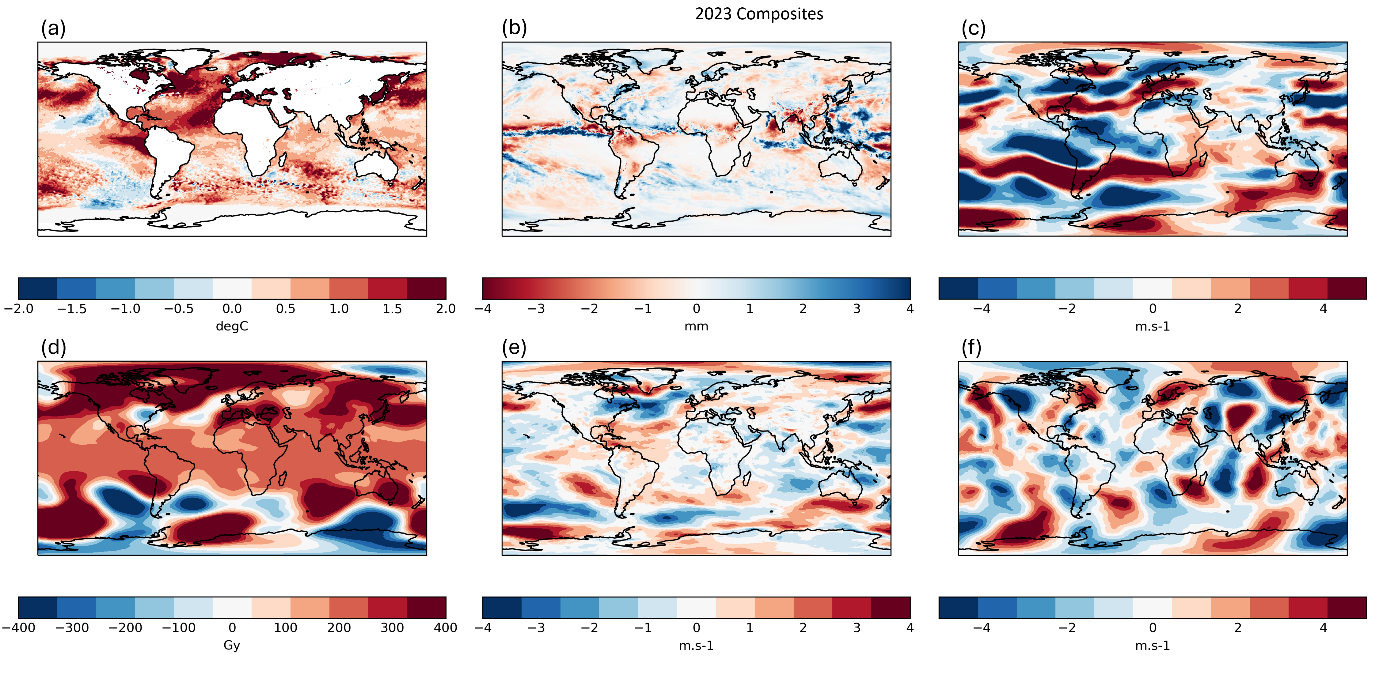


Fig. S5: Composite of anomalies of global (a) SST (b) precipitation (c) zonal wind at 200 (d) geopotential height at 500 hPa (e) zonal wind at 850 hPa (f) meridional wind at 200 hPa in JJA 2023, compared to the mean of El Niño years in 1950 – 2023 (n = 25), based on ERA5 reanalysis.


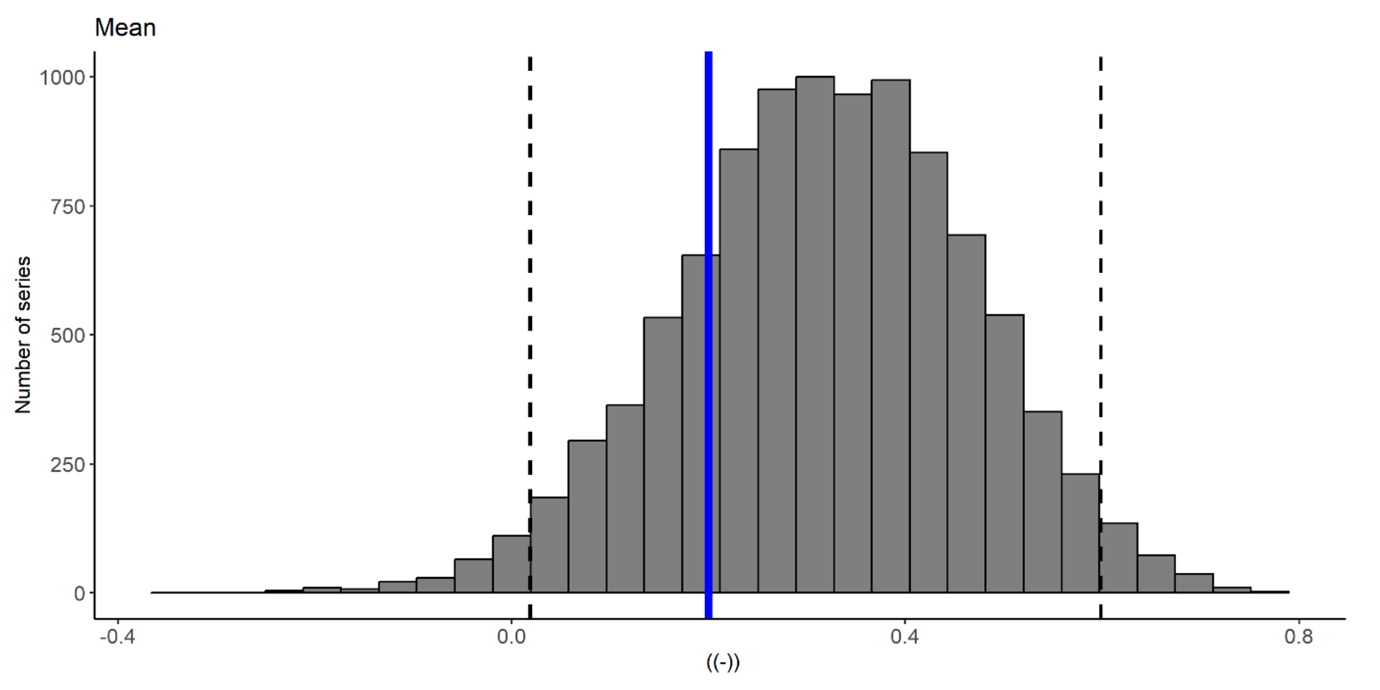


Fig. S6: Fidelity test of GloSea6 seasonal hindcast for the correlation between IOD and Borneo FWI in JJA. The blue solid line represents the observational estimate, the grey bars show the distribution of correlation coefficients between the subsampled (by 10,000 times) FWI and Dipole Mode Index, and the black dotted lines indicate the boundaries within 95% of the model distribution.
